# Observed Reductions in *Schistosoma mansoni* Transmission from Large-Scale Administration of Praziquantel in Uganda: A Mathematical Modelling Study

**DOI:** 10.1371/journal.pntd.0000897

**Published:** 2010-11-23

**Authors:** Michael D. French, Thomas S. Churcher, Manoj Gambhir, Alan Fenwick, Joanne P. Webster, Narcis B. Kabatereine, Maria-Gloria Basáñez

**Affiliations:** 1 Schistosomiasis Control Initiative, Imperial College London, London, United Kingdom; 2 Department of Infectious Disease Epidemiology, Imperial College London, London, United Kingdom; 3 Vector Control Division, Ministry of Health, Kampala, Uganda; Yale University, United States of America

## Abstract

**Background:**

To date schistosomiasis control programmes based on chemotherapy have largely aimed at controlling morbidity in treated individuals rather than at suppressing transmission. In this study, a mathematical modelling approach was used to estimate reductions in the rate of *Schistosoma mansoni* reinfection following annual mass drug administration (MDA) with praziquantel in Uganda over four years (2003–2006). In doing this we aim to elucidate the benefits of MDA in reducing community transmission.

**Methods:**

Age-structured models were fitted to a longitudinal cohort followed up across successive rounds of annual treatment for four years (Baseline: 2003, Treatment: 2004–2006; n = 1,764). Instead of modelling contamination, infection and immunity processes separately, these functions were combined in order to estimate a composite force of infection (*FOI*), i.e., the rate of parasite acquisition by hosts.

**Results:**

MDA achieved substantial and statistically significant reductions in the *FOI* following one round of treatment in areas of low baseline infection intensity, and following two rounds in areas with high and medium intensities. In all areas, the *FOI* remained suppressed following a third round of treatment.

**Conclusions/Significance:**

This study represents one of the first attempts to monitor reductions in the *FOI* within a large-scale MDA schistosomiasis morbidity control programme in sub-Saharan Africa. The results indicate that the Schistosomiasis Control Initiative, as a model for other MDA programmes, is likely exerting a significant ancillary impact on reducing transmission within the community, and may provide health benefits to those who do not receive treatment. The results obtained will have implications for evaluating the cost-effectiveness of schistosomiasis control programmes and the design of monitoring and evaluation approaches in general.

## Author Summary

Schistosomiasis is a parasitic disease of enormous public health importance, infecting over 200 million people worldwide, of which the large majority live in sub-Saharan Africa. Control programmes based on the mass treatment of individuals in infected areas with the drug praziquantel have been shown to be successful in reducing the parasite burden and likelihood of developing morbidity in those individuals who receive treatment. Using data from an ongoing intestinal schistosomiasis control programme in Uganda and through the application of a mathematical model, we show that an additional benefit of mass treatment is a decrease in parasite acquisition, via a reduction in the number of transmission stages in the environment. This leads to a lower rate of infection and reinfection of individuals in those areas. We show that this result is valid in areas of differing average infection intensity. The importance of this finding is that this will benefit untreated as well as treated individuals, and will allow a fuller estimation of the benefits of schistosomiasis control programmes.

## Introduction

**Figure 1:**
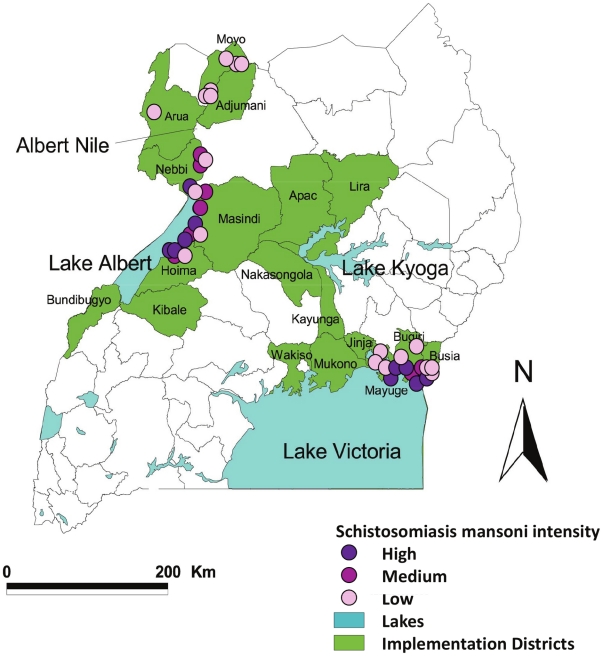
Baseline schistosomiasis prevalence in Uganda. Map of Uganda showing results from baseline prevalence mapping of intestinal schistosomiasis in the country. The three main areas of schistosomiasis transmission are situated along the shores of Lake Victoria, Lake Albert, and Albert Nile. Monitoring and evaluation (M&E) areas were chosen using a statistical sampling framework to provide a representative sample of the whole treated area described in [31]. The three levels of *Schistosoma mansoni* endemicity at baseline are represented by closed circles: high (≥400 epg, violet); medium (100–399 epg, purple), and low (1–99 epg, pale pink) transmission. Figure reproduced with permission from Zhang *et al*. (2007) [31].

In terms of its socioeconomic impact upon the afflicted populations, schistosomiasis constitutes the world's most important parasitic disease after malaria, infecting 207 million people worldwide, of whom 85% live in Africa [1]. The Schistosomiasis Control Initiative (SCI) was established in 2002 with the aim of helping establish sustainable schistosomiasis control programmes based on large-scale praziquantel (PZQ) administration. Uganda was the first country where SCI implemented control (and hence the area upon which this study focuses), capitalizing on the strength of its national expertise. In Uganda, intestinal schistosomiasis (caused by *Schistosoma mansoni*) is widespread, with highly endemic foci of infection around the waterbodies of Lake Albert, Lake Victoria, Lake Kyoga and along the Albert Nile (Figure 1). As there is very limited urinary schistosomiasis (due to *S. haematobium*) in Uganda, we focus on *S. mansoni* infections here. Morbidity is often caused by eggs rupturing the intestinal wall leading to blood loss and subsequent anaemia, and the immune response to eggs that become trapped in organs and tissues, leading to the development of hepatomegaly, splenomegaly, and eosinophilia [2].

*S. mansoni* adult worms reproduce sexually in humans, with eggs released with faeces into fresh water, where they can hatch, with free-living miracidia subsequently infecting a suitable freshwater (*Biomphalaria*) snail intermediate host, within which asexual reproduction occurs. Cercariae are then released by the snail back into water, which complete the life-cycle by infecting humans who come into contact with infested water. The infection in the (human) definitive host can be treated effectively with PZQ, a safe, affordable, and efficacious drug which kills the adult worms and therefore reduces egg counts. Although effective at clearing worm infections (with a cure rate efficacy of 50–80% and an egg reduction rate of 95% [3], [4], reinfection will occur following treatment (unless this is provided regularly for prolonged periods), and so, schistosomiasis control programmes based on chemotherapy have been aimed primarily at controlling morbidity rather than at suppressing transmission.

Given this reinfection and the absence of direct multiplication within the human host, the severity of infection (and hence of morbidity) is likely to reflect the cumulative exposure of an individual to infection over a period of years [5], in addition to the operation of individual host immune responses and concomitant immunity via already-established worms. The rationale of control programmes is that by reducing worm burdens in humans, and particularly children (who are the most likely to be heavily infected), the more serious sequelae of infection (organomegaly and fibrosis) are less likely to develop, and are more easily reversed if they do develop [6].

There are many examples of significant success in controlling infection intensity and morbidity in schistosomiasis control programmes using a mass drug administration (MDA) approach [7], [8], [9], [10]. What has not yet been quantified is the benefit of large-scale MDA to the wider community, including to those who are untreated, via reductions in environmental transmission. Such reductions would manifest as a decreased force of infection (*FOI*), the rate at which new incoming worms establish in the human host population. By estimating any change in the *FOI* under the chemotherapeutic pressure of such large-scale MDA we aim to assess the collateral impact of the programme on the untreated, as well as treated, sections of the population. Any reductions in the *FOI* observed would likely lead to lower infection intensities and a lower likelihood of developing subsequent morbidity.

The SCI's extensive monitoring and evaluation (M&E) framework is detailed elsewhere [7], [11], [12]. Briefly, in East Africa, school-age and community cohorts are monitored prior to each annual round of chemotherapy in order to quantify the impact of the programme on the prevalence and intensity of infection, and prevalence of disease. Parasitological data are recorded as the mean number of eggs per gram of faeces (epg) calculated from two Kato-Katz preparations from a stool sample to identify and count schistosome eggs as an indirect measure of worm burden [13], [14]. This approach was used throughout the programme in order to standardise results.

In this paper, we fit an age-structured mathematical model to longitudinal parasitological data collected over three rounds of PZQ treatment from locations with different baseline endemicity levels. The mathematical model is used to estimate changes in the *FOI* caused by MDA within the monitored cohort. These results are then used to predict the impact of MDA on other sections of the community which do not form part of the SCI monitoring programme, such as the general treated population (i.e. across all age ranges) and the untreated school-age population.

## Methods

### Age-Profiles of Infection

Schistosome infections tend to display a distinctive age-infection profile, with prevalence and intensity rising sharply in young children, peaking in adolescents and young adults (15–20 years), and declining in older age groups [15]. This relationship is thought to be caused by behavioural practices (age-specific changes in water contact and hygiene), and/or in combination with the gradual development of acquired protective immunity ([16] for urinary schistosomiasis; [17] for intestinal schistosomiasis). For this reason, and because of logistic advantages and the demography of populations, school-age children are often targeted for MDA in control programmes. Fitting models to this characteristic age-intensity profile at baseline is important to ensure that the model captures the relevant epidemiological characteristics at endemic equilibrium, prior to implementation of treatment.

The majority of mathematical models of schistosomiasis epidemiology and control have been fitted to baseline (pre-treatment) data only, with projections then being made of the likely impact of control interventions [18], [19], [20], [21]. These projections should be treated with caution as models fitted to baseline data may accurately reproduce pre-treatment patterns but can fail to fully capture post-treatment population dynamics of the parasite [22], (though see [23]). Part of the reason for this is the lack of data available to parameterise such longitudinal models. Due to the extensive size and longitudinal nature of the SCI cohorts, we have been able to fit the model simultaneously to pre- (baseline) and post- (three subsequent years) treatment data on infection and reinfection collected in areas differing in baseline infection intensity. Fitting to multiple years concurrently is expected to provide a more robust picture of the effects of treatment on parasite populations and help to measure any reductions in environmental transmission with multiple treatment rounds.

### Datasets

The cohorts were assembled from the SCI's M&E component of the Ugandan national schistosomiasis control programme, which commenced large-scale treatment with PZQ in 2003, and re-treated host populations at yearly intervals for 3 years (follow-up year 1 (F1), 2004; follow-up year 2 (F2), 2005; and follow-up year 3 (F3), 2006). A longitudinal cohort of school-age children who were positively identified and presented at each of the 4 treatment occasions (using unique identification codes) was constructed. This cohort consisted of 1,764 individuals (49.6% female), aged between 6 and 15 years. Additionally, a cross-sectional cohort of children and adults (3,387 individuals) treated at baseline (composed of 2,538 children ≤15 years of age (45.5% female) and 849 adults (46.8% female)) was constructed in order to provide age-specific infection profiles. The SCI treatment approach is to target (enrolled and non-enrolled) school-age children in all areas where schistosomiasis is endemic. In addition, where infection levels are high (as indicated by a prevalence of over 50% in school-age children), treatment of adults in the community is also carried out [11], [24]). Thus the overall treatment coverage in an area will vary depending on that area's underlying level of endemicity.

In order to explore the influence of the level of initial endemicity on model outputs, reductions in the *FOI*, and possible future treatment strategies, the model described below (see Current Mathematical Model) was fitted separately for areas where the average intensity of infection recorded at baseline (as the arithmetic mean epg across all hosts in the population) fell within the high, medium, or low categories proposed by the World Health Organization, i.e., heavy infection: ≥400 epg; moderate infection: 100–399 epg; light infection: 1–99 epg [24]. These categories were chosen as they are thought to relate to the likelihood of individuals developing morbidity [25]. Also, as the relationship between overdispersion in the distribution of egg counts per host and mean infection intensity differed sufficiently between areas, it was deemed that the model warranted separate fits (see Supplementary Information Protocol S1 and Figure S1).

The sizes and characteristics of the datasets for the longitudinally followed and cross-sectional cohorts in each of the three epidemiological settings are given in Table 1.

**Table 1:** Cohort sample sizes.

Intensity area (categorised by mean epg at baseline)	Baseline Total (Longitudinal; Cross-Sectional)	F1	F2	F3
	(2003)	(2004)	(2005)	(2006)
High (≥400)	1,210 (428; 782)	404	435	262
Medium (100–399)	1,613 (404; 1,209)	403	413	235
Low (1–99)	2,305 (909; 1,396)	851	916	600

Sample sizes for the baseline longitudinal and cross-sectional cohorts followed up across all four years in each of three endemic areas for *Schistosoma mansoni* in Uganda.

NOTE: Areas are classified according to their baseline intensity of infection (measured as the mean number of eggs per gram of faeces, epg, from all individuals sampled in that area). Numbers vary between years in the longitudinal cohort because some individuals presented on the day of treatment but did not provide faecal samples; individuals were included in the study from follow-up years as long as they were administered all 4 rounds of treatment. F1 = follow-up year 1, F2 = follow-up year 2, F3 = follow-up year 3.

### Descriptive Statistics

Arithmetic means of infection intensity were used as measures of central tendency [26], [27] in order to compare the means of age-groups and to provide agreement with the model output. Ninety five percent confidence intervals (95%CI) around the observed means were calculated via the normal approximation for large sample sizes [28]. Point estimates of prevalence of infection and categories of infection (as described above) were also calculated and the normal approximation to the binomial distribution was used to estimate their 95% CI given the large sample sizes available [28]. When preparing age-intensity profiles, age ranges were chosen to ensure a minimum of 20 individuals in each age-group. Given these relatively small sample sizes, instead of assuming a certain distribution of data, 95%CI were derived from 100,000 bootstrap re-sampling of the data with replacement [29].

Statistical comparison of means was performed via normal distribution *z*-tests for large samples (see Table 1). Prevalence values were compared by z-tests on the difference between two proportions [28]. The proportional reduction over treatments of infection prevalence and intensity was estimated as the absolute value of the ratio of the difference between the final and initial values to the initial value, expressed as a percent.

### Force of Infection (*FOI*)

The *FOI* for macroparasitic infections is defined as the *per capita* rate at which a host acquires new infections [30]. This can be interpreted in a number of different ways according to which stage of the parasites' life-cycle is of interest. The number of adult schistosomes within a host can rarely be measured directly, so parasitological surveys typically rely on faecal egg counts as a proxy for parasite intensity. Therefore routinely used diagnostic tools cannot identify newly established parasites until they reach patency and reproduce successfully. For the purposes of this paper the *FOI* is defined as the rate at which new incoming worms establish into adult parasites and reach patency (initiate detectable egg production) in the human host population. This may differ from the rate at which the host population is infected by other parasitic stages, such as by cercariae from the environment.

In order to measure any reductions in parasite establishment caused by the control intervention, we estimated the underlying *FOI* prior to treatment and following successive rounds of PZQ. One approach to measuring this reduction in infection would be to use parasitological information from previously untreated individuals entering the cohort each year (aged 6 yr in our case) as a proxy for the wider untreated population. Annual cross-sectional studies would then provide information on secular changes in infection markers, as done in previous studies [31]. This method estimates the reduction in the *FOI* over the lifetime of the 6-year old child. Though useful, this approach cannot be used to estimate the changes in the *FOI* after each round of treatment (a potentially more useful measurement due to its immediacy) since the results would be heavily influenced by assumptions regarding the amount of exposure to infective stages by infants and very young children. We have very little information about these children prior to their entry into the cohort. Previously, it has been assumed that very young children are not exposed and contribute little to contamination [32], [33]. However more recent work has reported higher infection prevalence levels than previously thought in very young children [34], [35]. Instead, we use a method of estimating the mean change in the *FOI* caused by each round of treatment by fitting the mathematical model to the rate of parasite reinfection observed in the longitudinal cohort. Parasite intensity estimates were made prior to treatment each year, so the change in the *FOI* (expressed as a ratio of the *FOI* at baseline) is calculated as an average of the rising value across the previous year. The reductions in the *FOI* seen after each round were assumed to be equal across ages, as studies have shown that the infection profile generally returns to the same pattern following reinfection [36].

### EpiSchisto

EpiSchisto [18], [37] is a deterministic model of schistosomiasis transmission based on partial differential equations which describe the rate of change in mean (adult) worm burden and immunity of the human hosts with respect to human host age and time. EpiSchisto has previously been fitted to baseline data and used to project the future course of control programmes in Tanzania and Ghana with mixed success [18], [19], [20]. A number of processes within EpiSchisto are very difficult to quantify separately and accurately. These include the contamination of the environment by the host population and the actions of host immunity, each of which we discuss in more detail in the next two sections. In past modelling studies, a range of plausible parameters have been used, and although they may not influence the fitting of equilibrium scenarios they will have a strong impact on post-control dynamics.

### Contamination of the Environment

EpiSchisto assumes that the relative contribution of each age group to the contamination of the environment is equal to its relative exposure to infective stages. There is very little evidence supporting this assumption due to the difficulty in measuring an individual host's contribution to transmission, which is likely to vary substantially according to local sanitation practices and environmental conditions. Overestimating the contribution to transmission of highly infected age groups will substantially overestimate the community benefits of chemotherapy, particularly of those programmes which target heavily infected age groups. Therefore the contamination function is not modelled explicitly here and we use the approach outlined below.

### Modelling Parasite Establishment

In EpiSchisto [37] and in previous modelling work [21], [38], the rates of infection and immunity have been modelled explicitly and separately with the rate of infection declining exponentially with acquired immunity according to the strength of such immunity. However, although it is widely accepted that there is some form of human acquired immunity to schistosome infections [15], [16], [17], [39], its mode of action, against which parasite stages it operates, how it is elicited, its strength, efficacy and duration *in vivo*, and whether acquired immunity is the chief explanation for the relative insusceptibility of adults, are all still incompletely understood [39], [40], [41]. Thus, there seems to be little justification for including an explicit immunity function in the model, given the likely correlation between parameters. For instance, there will be significant correlation between the length of immunological memory (i.e. the rate at which immunity is lost over time) and any changes in the *FOI* following chemotherapy.

### Current Mathematical Model

The approach taken in this paper is to model phenomenologically a composite *FOI* that incorporates together the rates of contamination, of parasite acquisition and the effects of any immunologically-mediated and/or host age-dependent processes that may modulate such a rate. This allows the number of new infections to be decoupled from the number of adult parasites, allowing the *FOI* to be estimated directly from data as opposed to being generated through the underlying assumptions of EpiSchisto (such as the highly uncertain contamination function). Given that our aim is to provide policy-orientated outputs rather than a detailed description of the underlying mechanisms of schistosomiasis transmission and infection, this should provide a more robust approach.

The rate of change in adult worm burden (*M*) with respect to host age (*a*) and time (*t*) can be written as the following immigration-death model,
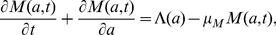
(1)where 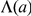 is the net *FOI* at age a, and 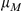 is the per worm death rate of established adult worms.

In turn, 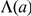 is given by Equation 2,
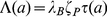
(2)where 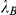 is the average underlying baseline *FOI* per person, 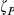 is the relative to baseline ratio of the average *FOI* after each round of treatment, with subscript *P* indicating the number of rounds of PZQ treatments the population has received, and the function 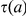 describes the (dimensionless) age-specific contact function normalized over the total host population which depends on two shape parameters (which together determine its convexity) (see Supplementary Protocol S2, equations S1 and S2). Thus, 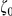 = 1 at baseline, and 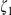, 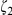, and 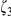 indicate the ratio of the *FOI* at follow-up years (F1), (F2), and (F3) relative to that at baseline, respectively. (A value of 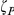 lower than 1 indicates a reduction in the *FOI* from baseline, and we consider a statistically significant reduction to be indicated where the entire range of the confidence interval lies below 1.) Λ(*a*) denotes the yearly average number of (egg-producing) worms acquired per person of age *a*. As such, this expression will comprise the product of the contact rate with infective stages, the probability of infection upon contact, and the average population of cercariae in the environment. It is assumed that treatment instantaneously reduces adult worm burden by 95% in all hosts given PZQ (the egg reduction rate [3], [4], see next section). In the longitudinal cohorts, therapeutic coverage is, by default, 100% as only those known to have received all four treatment rounds were included. A full list of model parameters is given in Supplementary Table S1.

### Fitting Approach and Sensitivity Analysis

The number of eggs per gram of faeces (epg) is thought in *S. mansoni* to be a reliable, indirect measure of the intensity of infection, particularly at the commencement of an intervention (though see [42] which suggests that egg production may be density dependent). Assuming that each worm produces on average 5.26 eggs per gram of faeces [18] allows the adult worm burden generated by the mathematical model in equation (1) to be converted to the epg count and fitted to the longitudinal data collected by the SCI.

The cross-sectional cohort at baseline consisted of both children and adults in order to provide the profile of age-related exposure. The age-stratified mean-based model was fitted simultaneously to the longitudinal cohort and cross-sectional baseline data using maximum likelihood estimation [43]. The method allows the model to be fitted to individual host data taking into account the high degree of parasite overdispersion observed (empirically described by the negative binomial distribution, see Protocol S1). This provides more robust estimates than those that can be achieved by fitting to aggregate measurements of infection intensity. The model was used to estimate concurrently the baseline *FOI*, 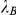, the two shape parameters of the contact function (*β* and *c*), and the change in the annual *FOI* after each round of chemotherapy (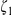, 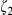 and 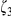) relative to that at baseline. In addition to these six parameters being fitted, a concurrent sensitivity analysis was carried out on fixed values of two further biologically important parameters with uncertainty around their values, namely, the per capita worm death rate (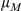), and the efficacy of PZQ (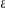) in terms of egg reduction rate expressed as a percentage). The fixed values used for the sensitivity analysis were taken from the literature and are presented in Supplementary Table S1.

The 6-dimensional parameter space was explored using the Latin Hypercube sampling method [44], [45]. As part of this fitting approach, the infection intensity observations of individuals were compared to the model-derived, age-specific mean intensity of infection. Ninety five percent confidence intervals around each of the model parameters were calculated using the Fisher Information Matrix [46] for a range of plausible adult worm mortality rates (from 2 to 10 years) and PZQ egg reduction efficacies (from 90% to 99%). Ninety five percent confidence intervals around model outputs were estimated by re-running the model and randomly selecting parameters from within their 95%CI bounds. Runs which generated likelihood values not statistically significantly different from the best fit run (tested using a χ^2^ distribution with the appropriate degrees of freedom) were used to construct 95%CI around model outputs [47]. The maxima and minima mean egg output at each timepoint from these included runs constituted the upper and lower confidence intervals respectively for the model output.

The longitudinal cohort consisted of individuals who were observed to have received all treatment rounds. As there was no replenishment of the youngest age classes it would be expected that without treatment there would be an increase in the intensity of infection in the cohort over the length of the study as the cohort ages. This is due purely to the increase in exposure typically experienced by children between the ages of approximately 5–15 years [23]. The fitted mathematical model takes this into account by allowing the age of the cohort to increase over time; not controlling for this would have led to underestimating the reductions in the *FOI*.

### Treatment Effects in the Broader Population

The model was used to investigate how temporal changes in the *FOI* caused by MDA may influence different sections of the population which are not part of the longitudinal cohort. It is assumed that any reduction in the *FOI* over successive rounds of chemotherapy is due to a reduction in the number of infective stages within the environment and not through secular changes in host immunity (which would mainly affect the treated population). The population age structure was estimated from Ugandan census data [48], assuming a constant human death rate with age (Figure S2). Estimates of the number of hosts in each of the different WHO intensity categories were generated using the relationship between infection prevalence and intensity that permitted estimation of the aggregation parameter of the negative binomial distribution as described in Protocol S1.

## Results

### Reductions in Intensity and Prevalence of Infection

Three rounds of treatment significantly reduced average infection intensity in all areas. In areas that were classified as of high endemicity at baseline, mean epg values fell by 84% (from 766 (95%CI: 704–828) epg at baseline to 121 (95%CI: 69–172) epg at follow-up year 3, *P*<0.001). In moderate endemicity areas, infection intensity fell by 75% (from 231 (95%CI: 209–257) to 58 (95%CI: 29–87) epg, *P*<0.001). In low endemicity areas there was an 87% decrease (from 33 (95%CI: 27–39) to 4.4 (95%CI: 1.6–7.1) epg, *P*<0.001). Significant reductions in infection prevalence were also observed, with decreases of 48% (from 84% (95%CI: 82–86) at baseline to 44% (95%CI: 38–50) at follow-up year 3, *P*<0.001); 55% (from 57% (95%CI: 55–59) to 25% (95%CI: 20–31), *P*<0.001), and 79% (from 21% (95%CI: 19–23) to 4.5% (95%CI: 2.8–6.2) *P*<0.001) in, respectively, high, moderate and low intensity areas.

### Age-Intensity Profiles of Infection at Baseline

**Figure 2:**
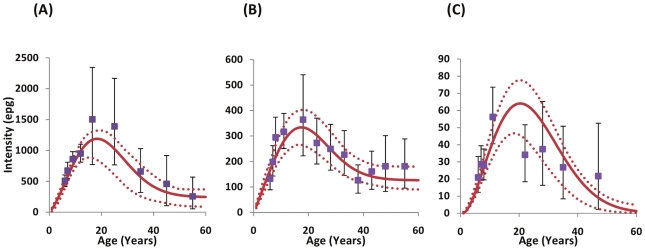
Age-profiles of *Schistosoma mansoni* infection intensity at baseline. Intensity is measured in eggs per gram of faeces (epg) at baseline as observed in the cross-sectional cohorts (blue markers) and fitted by the model (red line): A) high intensity areas; B) medium intensity areas; C) low intensity areas, as defined in Figure 1. Error bars are the 95% confidence intervals of the data calculated by 100,000 bootstrapping repetitions with replacement. Dotted lines are the 95% confidence intervals around model outputs. Age-groupings were chosen to ensure a minimum of 20 observations per category. Note the differences in the y-axis scales between the three endemicity levels.

The model successfully describes the baseline age-intensity profiles observed in the data from the three different endemicity levels, and replicates the classic convex relationship often seen in schistosome infections (Figure 2).

### Treatment Effects in the Treated Longitudinal Cohort

**Figure 3:**
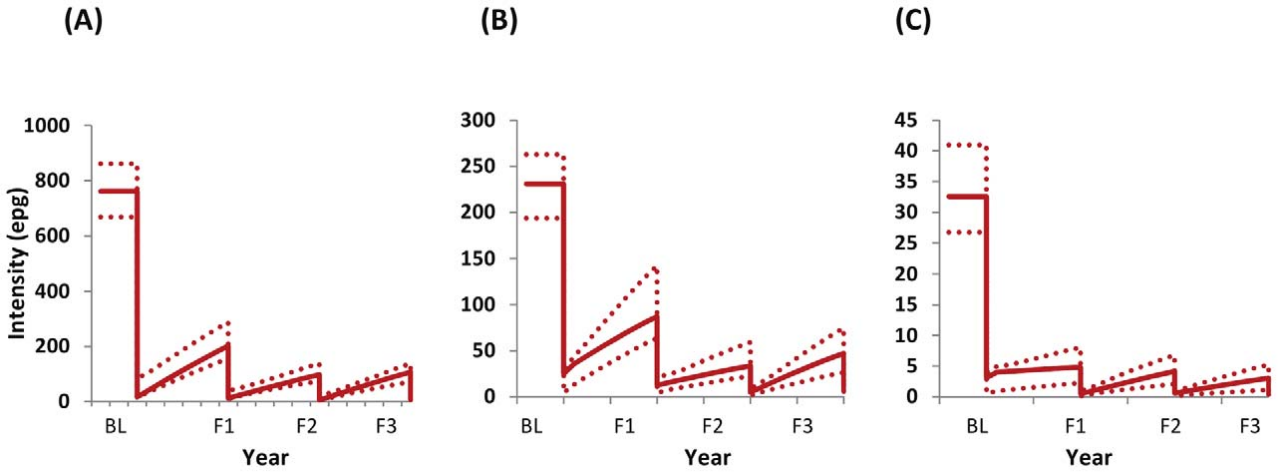
Impact of treatment on average infection intensity. The temporal dynamics of *S. mansoni* infection intensity (epg) in the longitudinal cohort in three areas of varying endemicity in Uganda after introduction of yearly treatment with praziquantel: A) high intensity areas; B) medium intensity areas; C) low intensity areas, as defined in Figure 1. The model was fitted to the baseline (BL) longitudinal and cross-sectional cohort data collected in 2003, and to the longitudinal cohorts for follow up years 1 (F1, 2004), 2 (F2, 2005), and 3 (F3, 2006) as described in the main text, Protocol S2, and Tables 1 and S1. Dotted lines indicate the 95% confidence intervals around the model outputs. Note the differences in the y-axis scales between the three endemicity levels.

The dynamics of infection intensity after the introduction of successive rounds of chemotherapy in the longitudinal cohort are shown in Figure 3.

Table 2 presents the estimated parameter values for the baseline *FOI* and for the relative to baseline reductions in the *FOI* that best fitted the data. In low intensity areas, where treatment is aimed only at school-age children, a substantial and statistically significant reduction (of about 76% from baseline) in the *FOI* was effected by a single round of MDA (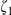 = 0.24). In moderate intensity areas (where there is a mixture of only school-age children treatment, and school-based and community treatment depending on an area's prevalence of infection), one treatment round resulted in no change in *FOI* (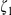 = 1.04), two treatment rounds achieved approximately a 66% reduction in *FOI* (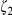 = 0.34), with a 54% reduction achieved after three rounds (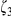 = 0.46), although the latter did not reach statistical significance (95% CI included 1). In high intensity areas (where both school-age children and the whole community receive MDA), the first treatment round only reduced the *FOI* by 22% from baseline (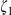 = 0.78) and this did not reach statistical significance. Subsequent reductions were of the order of 63% for the second (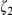 = 0.37) and third rounds of treatment (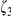 = 0.37), both of which were statistically significant.

**Table 2:** Best fitting parameter values.

Parameter	Description	High	Medium	Low
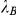	Baseline *FOI*	30.0 (10.2, 57.7)	10.0 (3.8, 20.6)	1.7 (0.3, 6.5)
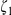	The proportion of *FOI* relative to baseline after 1 PZQ round	0.78 (0.46, 1.50)	1.04 (0.49, 2.00)	0.24 (0, 0.61)
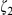	The proportion of *FOI* relative to baseline after 2 PZQ rounds	0.37 (0.22, 0.63)	0.34 (0.13, 0.74)	0.32 (0.06, 0.64)
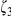	The proportion of *FOI* relative to baseline after 3 PZQ rounds	0.37 (0.19, 0.86)	0.46 (0.17, 1.06)	0.20 (0.04, 0.43)

These values are estimated separately for areas of High, Medium, and Low average infection intensity at baseline. Parameter 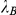 is the force of infection at baseline (average number of incoming worms establishing and egg-shedding per person per year); 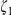 is the *FOI* relative to baseline following 1 round of PZQ treatment; 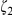 is the *FOI* relative to baseline following 2 rounds of treatment; 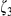 is the *FOI* relative to baseline following 3 rounds of treatment. Thus any figures below 1 indicate a reduction in the *FOI*. Figures in brackets are 95% CI calculated using the Fisher Information Matrix.

### Reductions in Heavy Infection in the Cohort and Wider Population

**Figure 4:**
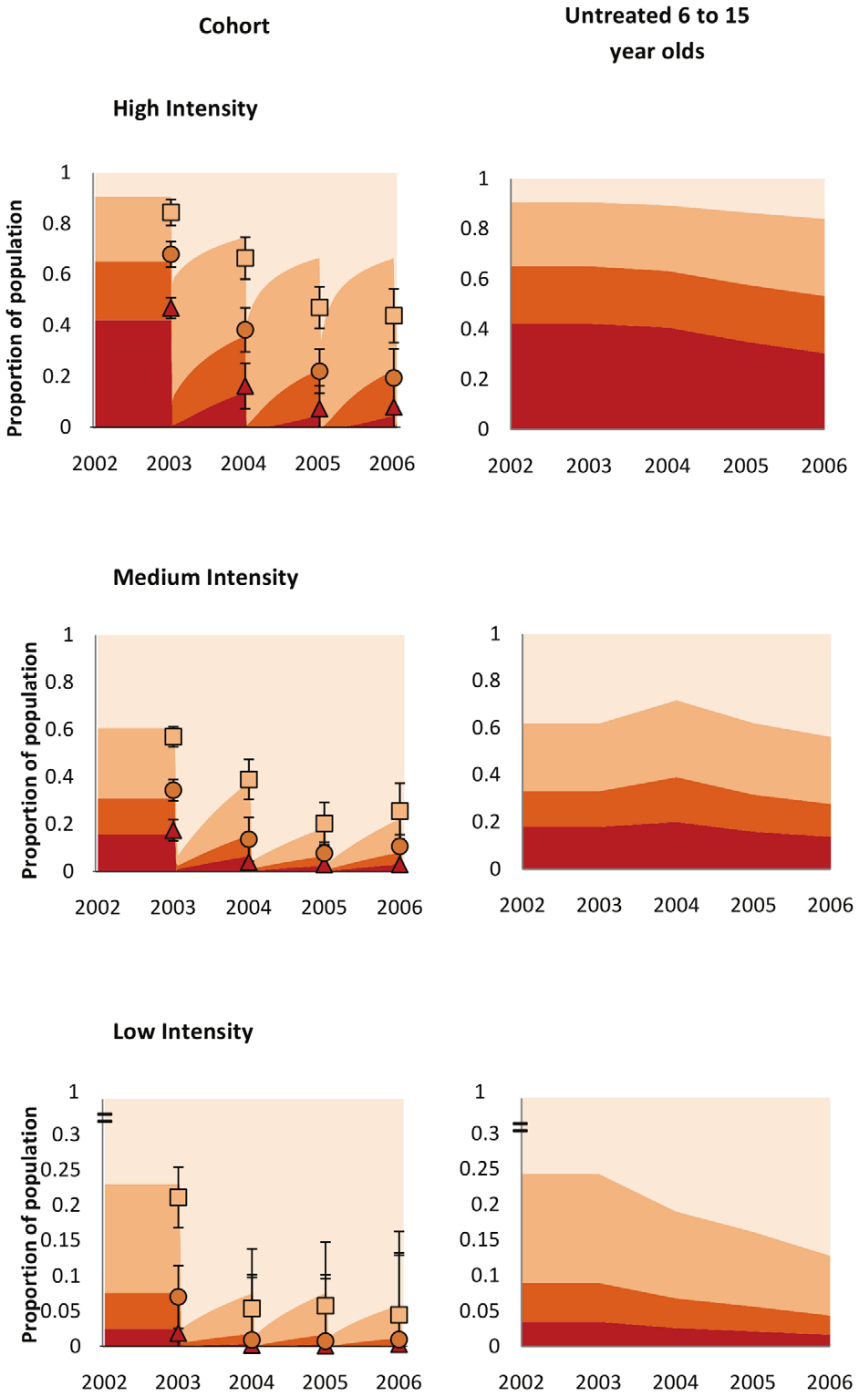
Impact of treatment on categories of infection intensity in treated and untreated populations. This shows the change in the proportion of people within each infection intensity category following praziquantel treatment. The left-hand column shows the observed figures from the baseline longitudinal and cross-sectional cohorts (data points and error bars representing 95% confidence intervals derived from 100,000 bootstrap repetitions with replacement) compared to the model-derived values (shaded areas). The right-hand column shows the predicted reduction in categories of infection intensity in those school-age children who do not receive treatment, using Uganda-specific demographic structure. Thus, any reduction in the prevalence of heavy infection in the latter will be due to changes in the *FOI*. Red = heavy infection (≥400 epg); dark orange = moderate infection (100–399 epg); light orange = light infection (1–99 epg), pale yellow = uninfected (0 epg). The upper, middle, and bottom rows refer, respectively, to areas of high, medium, and low intensity at baseline, as defined in Figure 1. The years correspond to: 2002–2003: baseline; 2004: follow up year 1; 2005: follow up year 2; 2006: follow up year 3.

A reduction in the numbers of people harbouring heavy infection (and who will thus be most likely to suffer from current and future morbidity) is clearly paramount from a morbidity control programme perspective. In high intensity areas the prevalence of heavy infection (proportion of individuals excreting ≥400 epg) fell from 47% (95%CI: 43–51) at baseline to 8% (95%CI: 0–19) after 3 rounds of treatment (a reduction of 83%, *P*<0.001). Similarly in areas with an average moderate intensity at baseline (100–399 epg), the percentage of individuals harbouring heavy intensity infections fell from 17% (95%CI: 13–22%) to 3% (95%CI: 0–15%) (a reduction of 83%, *P*<0.001). In areas of low intensity at baseline (<100 epg), the prevalence of those harbouring heavy infection fell from 1.91% (95%CI: 0–6.0%) to 0.33% (95%CI: 0–8.3%); a fall of 82.5% (*P* = 0.01). Figure 4 shows the reductions in the frequency of infection category in the different areas. The left-hand column demonstrates the fit of the model to observed frequencies of infection category in the longitudinal cohort. Parameter values obtained from fitting the model were then used to make predictions regarding the effect of MDA on the untreated human population. Using Uganda-specific demography, the impact of treatment on those school-age children (aged 6–15 yr) who did not receive treatment is shown in the right-hand column. Any changes here are caused purely by reductions in *FOI*.

### Reduction in Worm Acquisition

In areas of high intensity, the worm acquisition rate in school-aged children fell from 53.8 worms per person per year at baseline, to 19.8 following 3 rounds of treatment. Similarly in moderate intensity areas, the per capita worm acquisition rate falls from 15.9 to 7.3 per year, and from 2.7 to 0.5 per year in low intensity areas.

### Use of 6-Year-Olds to Measure Reductions in the *FOI*

**Figure 5:**
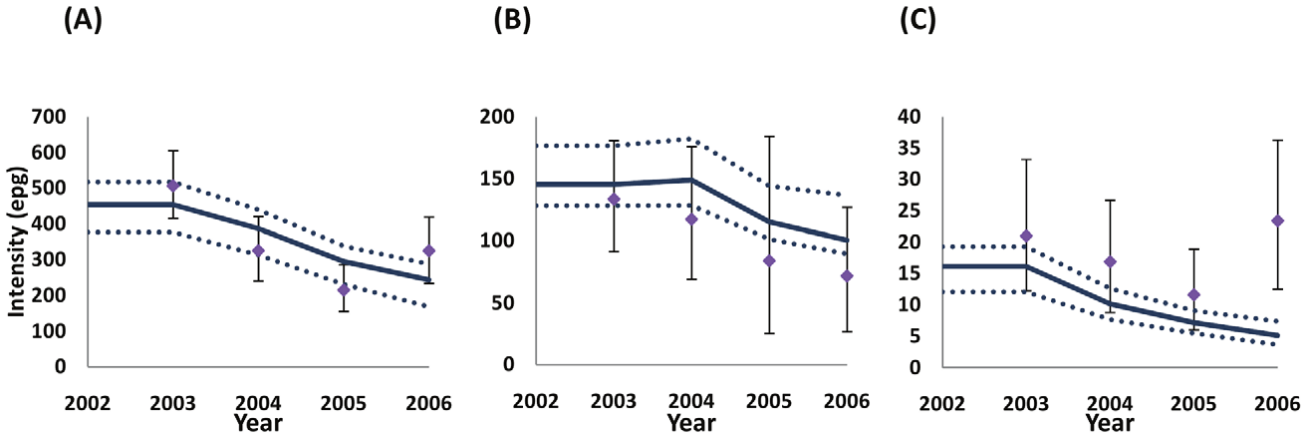
Model outputs for 6yr olds versus observed data. The comparison between the model temporal dynamics for 6-yr olds (solid lines) and the observed infection intensities of untreated 6-yr olds as they enter the cohort each year (data points): A) high intensity areas; B) medium intensity areas; C) low intensity areas, as defined in Figure 1. Dotted lines are 95% confidence intervals around the model outputs. Error bars are 95% confidence intervals on the data, derived from 100,000 bootstrap repetitions with replacement. Note the differences in the y-axis scales between the three endemicity levels. Years are as in Figure 4.

**Figure 6:**
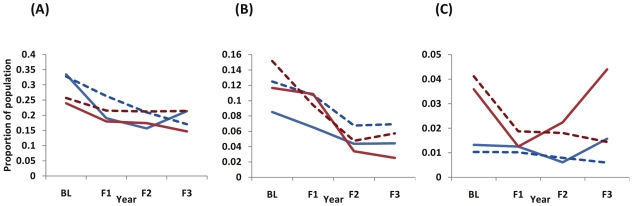
Categories of infection intensity in untreated 6yr olds. The comparison between model outputs (dashed lines) and observed values (solid lines) with respect to the frequency of infection intensity categories in previously untreated 6-yr olds entering the cohort each year: A) high intensity areas; B) medium intensity areas; C) low intensity areas, as defined in Figure 1. BL, F1, F2 and F3 as in Figure 3. Red lines indicate heavy intensity of infection (≥400 epg), and blue lines indicate moderate intensity of infection (100–399 epg). Note the differences in the y-axis scales between the three endemicity levels.

Figures 5 and 6 compare with model predictions and for untreated 6-year olds, the observed parasite load and the percentage of children within each intensity category, respectively. There is a statistically significant reduction in infection intensity (*P*<0.001) in the 6-year olds between baseline and follow-up year 2 (F2) in areas which were classified as of high intensity at baseline, and non-significant declines for moderate (*P* = 0.324) and low intensity areas (*P* = 0.142). Conversely, the intensity of infection in 6-year old children is higher at F3 than at F2 in both the high (*P* = 0.051) and low (*P* = 0.093) intensity schools. Such a result may be interpreted as a reduction in population MDA coverage in the last round of treatment. However, our analysis indicates that this was not reflected in the rate of parasite reinfection in older age groups (Table 2). In high intensity areas the observed data and predicted outcomes match relatively well though in medium and low intensity areas the model over- and underestimates parasite intensity respectively. We derive a reasonable fit to the data for the change in frequency of heavy and moderate infection categories (light and non-infected are omitted for clarity).

## Discussion

The impact of many helminth control programmes is often underestimated if reductions in *FOI* are not calculated. This issue has been raised by Miguel and Kremer [49] who noted that following a schistosomiasis control programme in Kenya, the intensity of infection decreased in the treated populations and also, crucially, in a nearby untreated population. The study presented here demonstrates that, as well as reducing the intensity of infection, MDA has a substantial impact on reducing the rate of parasite establishment in the human host population in some areas, even after one treatment round (and without reaching universal coverage). Although the magnitude of the reduction in the *FOI* varied in the three endemicity levels investigated, there were significant reductions in all areas. Quantifying these reductions is important from a programmatic point of view, and because these results can be used to project the dynamics of infection in the general population.

For the purposes of this paper the *FOI* was defined as the rate of establishment of patent infections. Other interpretations of the *FOI* are possible, such as the rate of cercarial acquisition by humans (leading or not to successful parasite establishment). However the current definition can be regarded as the most closely linked to the likelihood of developing morbidity as disease sequelae are related to the production of schistosome eggs and the host's corresponding immune response, with the production of eggs proportionally related (linearly or otherwise) to the adult worm burden [18], [30], [42].

Our current lack of understanding as to how host immunity influences the rate of host reinfection suggests that the changes in the *FOI* over time need to be interpreted carefully. The reduction in the rate of parasite reinfection following chemotherapy could be explained either through a decrease in environmental transmission or a decrease in the treated host population's susceptibility to reinfection. PZQ treatment releases somatic parasite antigens (not otherwise exposed to the host's immune system) which may elicit protective responses that facilitate resistance to reinfection [50], [51], [52]. However, it is far from clear how much this immunological response may influence the rate of *S. mansoni* establishment, development, or fecundity. The significant reduction in the intensity of infection in the untreated 6-year olds between Baseline and Follow-up Year 2 in high intensity areas provides evidence that there is possibly a true decrease in the number of infective stages within the environment following chemotherapy. These children had never received treatment so would not have had their immune system boosted by schistosome antigens revealed after PZQ treatment. Indeed, if PZQ treatment reduced significantly the rate of reinfection, the model would be expected to underestimate parasite intensity in untreated 6-year olds. The most extreme manifestation of this would be in the high intensity areas, where higher parasite burdens would lead to greater release of immunogenic antigens with possibly an elevated degree of immunological resistance. This would in turn cause the reduction in the *FOI* to be overestimated and the observed intensity in 6-year olds to be higher than model predictions. Figures 5 and 6 indicate this is generally not the case, especially not in high intensity regions.

However, the increase in intensity levels in 6yr olds between F2 and F3, a change which is not reflected in the wider population, highlights the dangers of relying too heavily on a single age group (with potentially low sample size) to detect secular changes in parasite exposure. There is likely to be considerable uncertainty about the exposure and infection patterns of pre-school age children, and potentially significant variance between different settings (i.e. related to proximity to water source). Currently there are very little data available on the profiles of exposure and intensity for infants and young children (<6yrs particularly) which would help highlight this issue. In addition, as these data begin to become available the contact function in the model (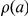 See Equation S1) could be updated as this would have a significant impact on estimating changes in the *FOI*.

The greatest relative reductions in the *FOI* after one round of treatment were observed in low intensity areas. This is perhaps not surprising given that the underlying intensity of transmission in this area is probably lower, resulting in a weaker resilience to control perturbations. However, significant reductions in the *FOI* were also observed in medium and high intensity areas after two rounds of treatment, and the *FOI* remained suppressed with a third round. There was only a modest decrease in the *FOI* in areas of high intensity and no reduction in medium intensity areas following the first round of treatment. Whether this is due to low coverage of the wider MDA programme in the region in the first year is difficult to ascertain given the well documented difficulties of accurately measuring actual population coverage in these programmes [53]. Multiple rounds of treatment could be required to reduce environmental contamination of the parasite in medium and high intensity areas. Transmission may be more resilient in these areas, and this may be caused by a relaxation in density-dependent processes acting on the parasite infra-populations (within-definitive host populations) following a treatment that kills adult worms and hence reduces parasite density. Such processes have been documented affecting the rate of parasite establishment in helminths (for onchocerciasis see [54]; for schistosomes [55], [56], with the latter exploring the relationship between transmission and virulence, which may be related to density dependence). The differences observed in the relative *FOI* reductions between areas of different endemicities may also be related to secular changes in environmental transmission in these areas, such as the extent of suitable water-contact sites, and/or the distribution of the snail intermediate host. It should be remembered that in all areas, without further sustained intervention, the reductions in the *FOI* will only be transitory and will return towards pre-treatment levels.

The results presented here indicate that the SCI programme is achieving significant reductions in *S. mansoni* infection intensity in treated individuals in all areas, and also in untreated individuals in high intensity areas in Uganda. The consecutive reductions in the proportion of those heavily infected in the population, principally in areas of high baseline endemicity are particularly striking, especially as this subset of individuals is thought to be the most likely to develop schistosomiasis-associated morbidity later in life [4]. Whether this constitutes an ‘important’ reduction in infection intensity is an interesting question. Clearly, much still remains to be ascertained as to the precise relationship between infection intensity and host morbidity. However it is clear that high infection intensities, particularly in childhood, are associated with increased subsequent chronic morbidity [2], [57], [58]. Therefore it is logical to predict that reducing infection intensities from ‘heavy’ to ‘medium’ as classified by the WHO represents a meaningful reduction (see Figure 4).

By extension, the importance of reducing the *FOI* is also demonstrated in Figure 4 (right hand column particularly). Here we can observe a reduction in the proportion of untreated individuals harbouring heavy intensities of infection. It may be expected that this would also result in a reduction of the morbidity in those individuals in future years.

Traditionally, MDA is viewed as a short- to medium-term solution to controlling schistosomiasis morbidity, whilst aiming for longer-term interventions such as improved sanitation and increased access to clean water to reduce transmission. Given the need to optimize interventions in resource-constrained settings, designing the most efficient and cost-effective control programmes is crucial. Programmatic costs can be lowered by reducing MDA frequency or through a shift towards targeted treatment. In low intensity areas treatment targeted at school-age children appears to have reduced the *FOI* substantially, justifying this approach. However, further studies are required to determine whether targeted treatment can reduce the *FOI* in medium to high transmission intensity areas (since a combination of school and community treatment were used in these regions). One possible approach could be to switch to targeted treatment after a number of rounds of population-wide MDA have reduced the infectivity of the environment. However, any decision to reduce the population-wide coverage or to introduce intermittent “treatment holidays” should take into consideration how this would influence the community *FOI* and thus morbidity within the untreated section of the population. Ultimately, those making decisions on MDA frequency and breadth should consider how compliance varies over time across the population, to assess whether the extra cost of frequent population-wide MDA can be justified through reductions in environmental contamination.

This study explores the changes in the *FOI* with yearly treatment for *S. mansoni* in an East African setting. Further work could compare this with a biennial treatment approach such as those taking place in the SCI programmes in West Africa, and in the future to any programmes that operate a twice-yearly treatment approach, where data are available. Given that in this study the *FOI* is calculated across the year and is therefore actually an average of a rising value, it may be predicted that increasing treatment frequency to every 6 months will have a greater than additive effect in reducing the *FOI*.

This study has implications for the design of M&E programmes, the purpose of which are to record, measure, and interpret the impact of control interventions appropriately. Firstly, longitudinal studies following the same children annually should control for the ageing of the cohort, given the highly convex age-intensity and age-exposure profiles seen in schistosomiasis. Secondly, treatment is often aimed primarily at school-age children; however, it is important to understand the patterns of exposure and infection that are occurring in pre-school children [59], as these patterns are still open to some debate [32], [34]. Involving these younger age groups who do not receive treatment in the monitoring programme will improve our understanding of the secular changes in transmission caused by a morbidity control programme, helping differentiate between the impact of PZQ on the individual and the community.

The aim of the SCI is morbidity control and thus the SCI's M&E is designed to reflect changes in the human parasite component, rather than the snail intermediate host, and therefore a method for estimating changes in the *FOI* from the human parasite burden (as measured by epg) [22] is presented here. As such, to our knowledge this is the first study attempting to quantify changes in the *FOI* caused by a large-scale schistosomiasis control programme using routinely collected data. Other methods, such as monitoring infections in the vector, have been used to assess the impact of MDA on the transmission potential of the parasite. For example Sturrock and colleagues [60], [61] used long-term snail sampling and cercariometry, whilst Butterworth and colleagues [4] examined changes in incidence of new infections among young children. (See also Yaméogo and colleagues [62] for an example of the use of black fly infectivity to estimate changes in the transmission of human onchocerciasis caused by control programmes). These approaches, incorporating longitudinal snail sampling to capture seasonal variations in transmission [63], will likely provide ultimate confirmation of reductions in *FOI*, and will provide useful validation of the results obtained in the present study. Combining these different approaches and incorporating the impact of non-random patters of compliance in relation to parasite burden as has been suggested [64], [65], [66], [67], would provide valuable information on the true effectiveness of MDA control programmes. This work will also help identify areas that are potentially open to local elimination of the disease and of the infection reservoir, and we advocate that the use of mathematical models could guide this process.

### Significance and Conclusions

Quantifying the reduction in transmission of a schistosomiasis control programme helps to understand fully the benefits of MDA to the entire human population. This study provides one of the first attempts to quantify the impact of large-scale MDA on transmission and the *FOI*. The ancillary benefit of MDA on transmission can be used as a powerful advocacy tool aimed at those funding and implementing programmes. There is a need for similar studies to investigate the effect of MDA in other eco-epidemiological settings, and for other parasitic diseases where MDA is a key component of infection and morbidity control.

## Supporting Information

Appendix S1 References to supporting information.
(0.02 MB DOC)

Figure S1 Relationship between school-level prevalence of infection and average infection intensity (epg) fitted as described in Protocol S1. A) Areas that recorded high infection intensity (epg≥400) at baseline, B) areas that recorded medium infection intensity (100≤epg<400) at baseline, and C) areas that recorded low infection intensity (1<epg<100) at baseline. The schools were sampled at baseline (turquoise squares) and re-sampled at follow up year 1 [F1] (green diamonds), [F2] (pale pink circles), and [F3] (light blue triangles). Note changes in scale of axes.
(0.22 MB TIF)

Figure S2 Comparison of observed population age-structure of Uganda (source: U.S. Census Bureau [3]) and the model-derived age-structure fit assuming a constant death rate.
(0.19 MB TIF)

Protocol S1 (0.05 MB DOC)

Protocol S2 (0.29 MB DOC)

Table S1 Parameter definitions and values used in the model. The table differentiates between parameters that were fixed throughout and those that were fitted using baseline cross-sectional and longitudinal cohort data. H = Areas of high average intensity at baseline (≥400epg), M = Areas of medium average intensity at baseline (100–399 epg), L = Areas of low average intensity at baseline (1–99epg).
(0.07 MB DOC)
