# Supplementary material for: Observed Reductions in Schistosoma mansoni Transmission from Large-Scale Administration of Praziquantel in Uganda: A Mathematical Modelling Study
Source: PLoS Negl Trop Dis. 2010 Nov 23;4(11):e897. doi: 10.1371/journal.pntd.0000897 (PMC2990705; doi:10.1371/journal.pntd.0000897)
Supplement: Appendix S1 — References to supporting information. (0.02 MB DOC) [file pntd.0000897.s001.doc]

**References**

1. Chan MS, Anderson RM, Medley GF, Bundy DAP (1996) Dynamic aspects of morbidity and acquired immunity in schistosomiasis control. Acta Trop 62: 105-117.

2. WHO (2002) Prevention and control of schistosomiasis and soil-transmitted helminthiasis. Geneva: World Health Organization. i-v1 p.

3. United States Census Bureau (2005). U.S. Census Bureau International Data Base. http://www.census.gov/ipc/www/idb/country.php

4. Anderson RM, May RM (1991) Infectious Diseases of Humans: Dynamics and Control. Oxford: Oxford University Press.

5. Kumar V, Gryseels B (1994) Use of praziquantel against schistosomiasis: a review of current status. Int J Antimicrob Agents 4: 313-320.
