# Supplementary figures and images for: Observed Reductions in Schistosoma mansoni Transmission from Large-Scale Administration of Praziquantel in Uganda: A Mathematical Modelling Study
Source: PLoS Negl Trop Dis. 2010 Nov 23;4(11):e897. doi: 10.1371/journal.pntd.0000897 (PMC2990705; doi:10.1371/journal.pntd.0000897)

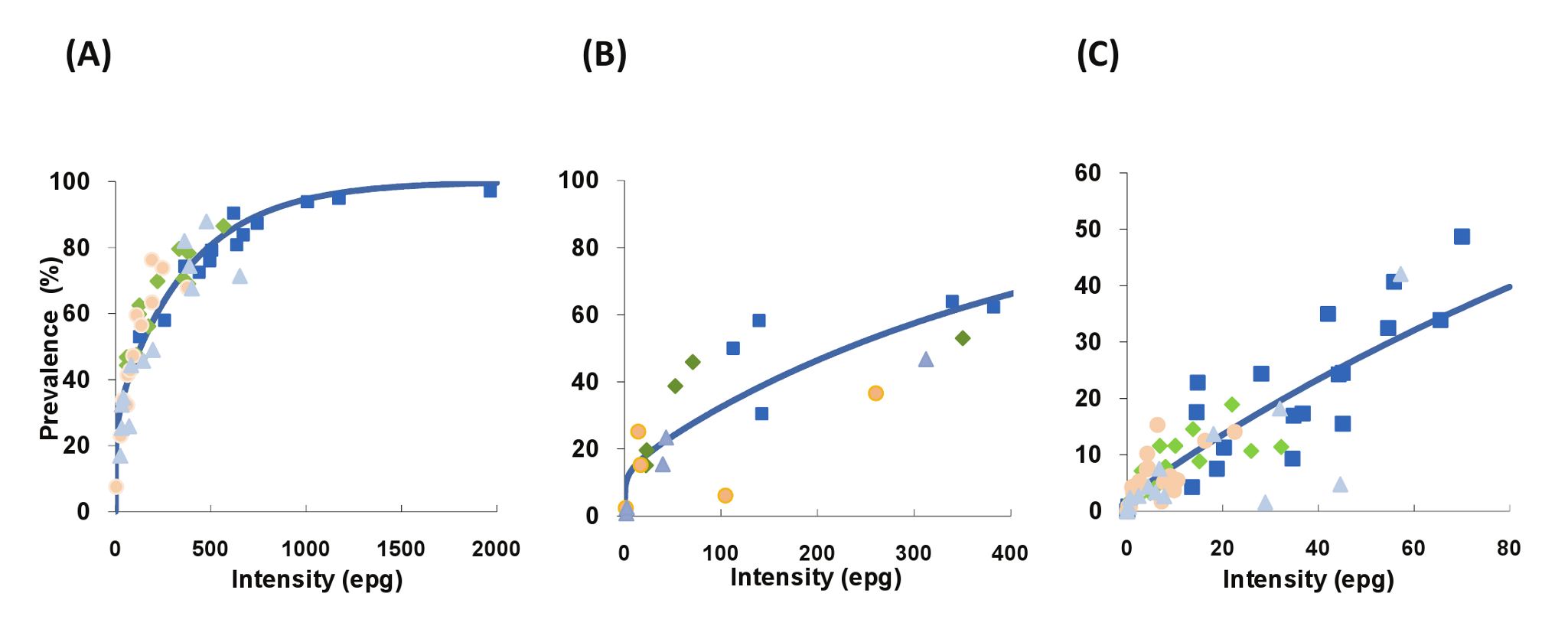

Supplement: Figure S1 — Relationship between school-level prevalence of infection and average infection intensity (epg) fitted as described in Protocol S1. A) Areas that recorded high infection intensity (epg≥400) at baseline, B) areas that recorded medium infection intensity (100≤epg<400) at baseline, and C) areas that recorded low infection intensity (1<epg<100) at baseline. The schools were sampled at baseline (turquoise squares) and re-sampled at follow up year 1 [F1] (green diamonds), [F2] (pale pink circles), and [F3] (light blue triangles). Note changes in scale of axes. (0.22 MB TIF) [file pntd.0000897.s002.tif]

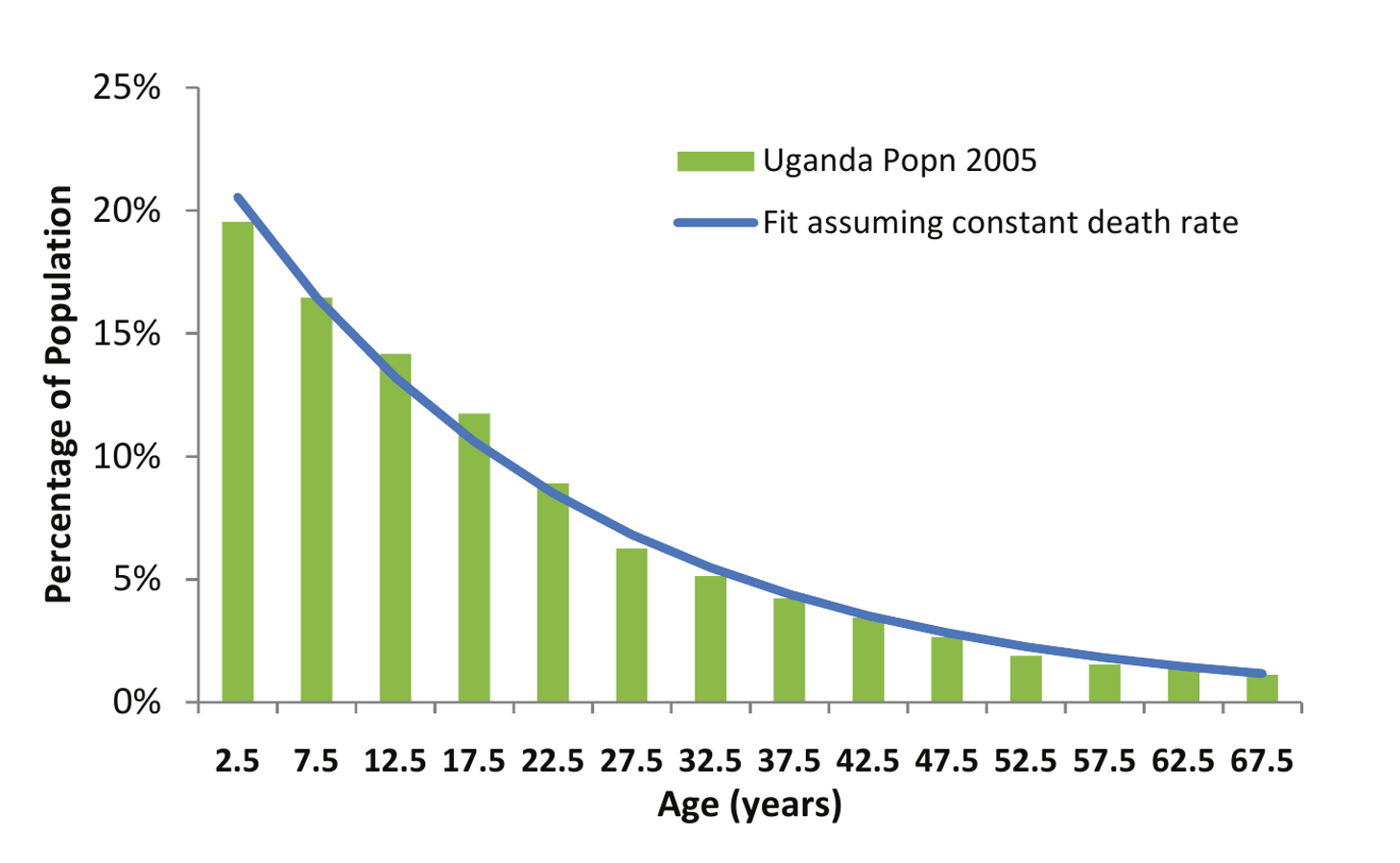

Supplement: Figure S2 — Comparison of observed population age-structure of Uganda (source: U.S. Census Bureau [3]) and the model-derived age-structure fit assuming a constant death rate. (0.19 MB TIF) [file pntd.0000897.s003.tif]
