# Supplementary material for: Observed Reductions in Schistosoma mansoni Transmission from Large-Scale Administration of Praziquantel in Uganda: A Mathematical Modelling Study
Source: PLoS Negl Trop Dis. 2010 Nov 23;4(11):e897. doi: 10.1371/journal.pntd.0000897 (PMC2990705; doi:10.1371/journal.pntd.0000897)
Supplement: Protocol S1 — (0.05 MB DOC) [file pntd.0000897.s004.doc]

**Protocol S1. Overdispersion**

Schistosomes typically display an overdispersed distribution in the human host population, whereby the majority of hosts are uninfected or lightly infected (harbour a low parasite burden), but a small minority are heavily infected (harbour a high infection intensity). This parasite aggregation within the host population is typically described using the negative binomial distribution (NBD) for convenience, which has two parameters: an inverse overdispersion parameter (*k*) and the arithmetic mean worm burden (*M*), as with the original EpiSchisto® model [1]. Here we estimate the overdispersion parameter by fitting by maximum likelihood the relationship between the infection prevalence (*P*) and the mean infection intensity (*M*, measured as epg, eggs per gram of faeces) that derives from assuming a NBD of epg among hosts, i.e., $P(M)=1-\left[1+\left(\frac{M}{k(M)}\right)^{-k(M)}\right]$, allowing the degree of parasite overdispersion to vary with mean worm burden according to $k(M)=k_{0}+k_{1}M^{k_{2}}$. This allows the functional form between the overdispersion parameter and the mean number of epg to take a constant value $\left(k_{0}>0; k_{1}=0\right)$, a linear expression $\left(k_{2}=1\right)$, a power function $\left(k_{2}\neq1\right)$, or a more complex form.

Because the relationship between *P* and *M* was found to differ between areas of high, medium and low average infection intensity at baseline, separate overdispersion parameters were calculated for each area. Within each area, however, the relationship between *P* and *M* appeared to be consistent before and after chemotherapy (Figure S1), which allowed the same functional form and parameters estimated for each area to be used at baseline, and follow-up years [F1], [F2] and [F3] (see main text and section below: Using the model to make predictions regarding the impact of reductions in the *FOI* on the untreated cohort of children aged 6 to 15 years).
