# Supplementary material for: Observed Reductions in Schistosoma mansoni Transmission from Large-Scale Administration of Praziquantel in Uganda: A Mathematical Modelling Study
Source: PLoS Negl Trop Dis. 2010 Nov 23;4(11):e897. doi: 10.1371/journal.pntd.0000897 (PMC2990705; doi:10.1371/journal.pntd.0000897)
Supplement: Protocol S2 — (0.29 MB DOC) [file pntd.0000897.s005.doc]

**Protocol S2. Mathematical model**

The full partial differential equation describing the change in the number of adult worms per host with respect to time and host age is given in equation [1] of the main text. For the purposes of fitting the model to the data, age is discretized in age classes (recorded in the field as integer age in years).

The unnormalized function that represents the relative contact rates distributed over age, denoted *ρ*(*a*), is described by the following equation [S1],

$\rho(a)=a e^{-\left(\beta a\right)^{2}}+c$ [S1]

with parameters *β* and *c* (where $c\geq0$) determining its humped relationship with host age, and *a* representing age class, ranging from 0 to 60 years.

In order to use this continuous function of age in the modelling process, it is discretized by partitioning the human population into *n* age groups each with width 1 year and mid value $a_{1}$ through to $a_{n}$ so that $n=60$and the upper age of the $n^{th}$age class is 60 years. The continuous function $\rho(a)$ then becomes $\rho(a_{i})$ where *i* denotes the *i*th age group.

In order to normalize this function, to render it dimensionless and to restrict its purpose to distributing contacts over age appropriately (information relating to the number of contacts per unit time will then be subsumed within $\lambda_{B}$, the baseline force of infection), we divide $\rho(a_{i})$ by a normalizing constant, which is the sum of the function $\rho(a_{i})$divided by the number of age groups, *n*. The resulting normalized contact function is denoted $\tau(a_{i})$ and is given by,

$\tau(a_{i})=\frac{\rho(a_{i})}{\frac{1}{n}\sum_{a_{i}=1}^{a_{i}=n} \rho(a_{i})}$ [S2]

**Using the model to make predictions regarding the impact of reductions in the *FOI* on the untreated cohort of children aged 6 to 15 years**

The human host population is assumed to have a constant death rate, with a mean life-expectancy ($1/\mu_{H}$) of 50 years and a maximum age, *a_n_* of 60 years (Figure S2). The proportion of hosts in age class *a_i_* is used to calculate the proportion of hosts in each age category of the untreated child population (6-15 years). This choice of age group is dictated by the design of the cohorts in the SCI. Note that we assume that the probability of being treated is independent of host age in this group given the scarcity of reliable and village-specific information about school absenteeism patterns. The model for the untreated cohort is identical to the one described in the main text for the treated cohort, and for both we use the estimated changes in the *FOI* to predict the temporal changes in parasite density. We then use the overdispersion functions to predict the proportion of hosts in each infection intensity category (as defined by the WHO [2]).

**Table S1.** Parameter definitions and values used in the model. The table differentiates between parameters that were fixed throughout and those that were fitted using baseline cross-sectional and longitudinal cohort data. H = Areas of high average intensity at baseline (≥400epg), M = Areas of medium average intensity at baseline (100-399 epg), L = Areas of low average intensity at baseline (1-99epg).

| **Parameter** | **Symbol** | **Units** | **Value [sensitivity analysis] or (95% CI)** | **Fixed or fitted parameter** | **Source** |
| --- | --- | --- | --- | --- | --- |
| Overdispersion parameter as a function of mean intensity | $k_{0}$ | Dimensionless | H: 0.091; M: 0.048; L: 0.001 | Fitted, Figure S1 | Protocol SI |
| $k(M)=k_{0}+k_{1}M^{k_{2}}$ | $k_{1}$ | Dimensionless | H: 0.0019; M: 0.0032; L: 0.0192 | Fitted, Figure S1 | Protocol SI |
|  | $k_{2}$ | Dimensionless | H: 1.000; M: 1.000; L: 0.667 | Fitted, Figure S1 | Protocol SI |
| Human mortality rate | $\mu_{H}$ | Year^–1^ | 0.0441 | Fitted, Figure S2 | [3] |
| Worm lifespan | $1/\mu_{M}$ | Years | 4 [2, 10] | Fixed | [4] |
| Drug efficacy | $\varepsilon$ | Dimensionless | 0.95 [0.90, 0.99] | Fixed | [5] |
| Baseline *FOI* | $\lambda_{B}$ | Year^–1^ |  | Fitted | Estimated in this paper |
| Ratio of *FOI* following PZQ treatment round *P* relative to baseline (*P* = 1, 2, 3) | $\zeta_{P}$ | Dimensionless |  | Fitted | Estimated in this paper |
| Shape parameter of the age exposure profile (contact function, equation [S1]) | $\beta$ | Year^–1^ | H: 0.052 (0.041, 0.113) M: 0.056 (0.039, 0.097) L: 0.040 (0.011, 0.065) | Fitted | Estimated in this paper |
| Shape parameter of the age exposure profile (contact function, equation [S1]) | $c$ | Years | H: 1.95 (0.37, 6.54) M: 4.30 (0.73, 6.93) L: 0.164 (0, 5.93) | Fitted | Estimated in this paper |

**Figure S1.** Relationship between school-level prevalence of infection and average infection intensity (epg) fitted as described in Protocol S1. A) Areas that recorded high infection intensity (epg ≥ 400) at baseline, B) areas that recorded medium infection intensity (100 ≤ epg < 400) at baseline, and C) areas that recorded low infection intensity (1 < epg <100) at baseline. The schools were sampled at baseline (turquoise squares) and re-sampled at follow up year 1 [F1] (green diamonds), [F2] (pale pink circles), and [F3] (light blue triangles). Note changes in scale of axes.

**(A)** **(B)** **(C)**


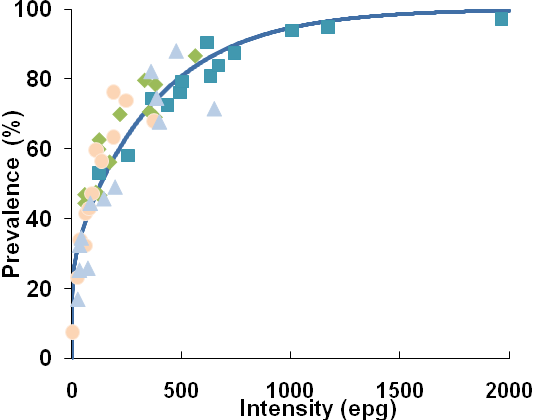

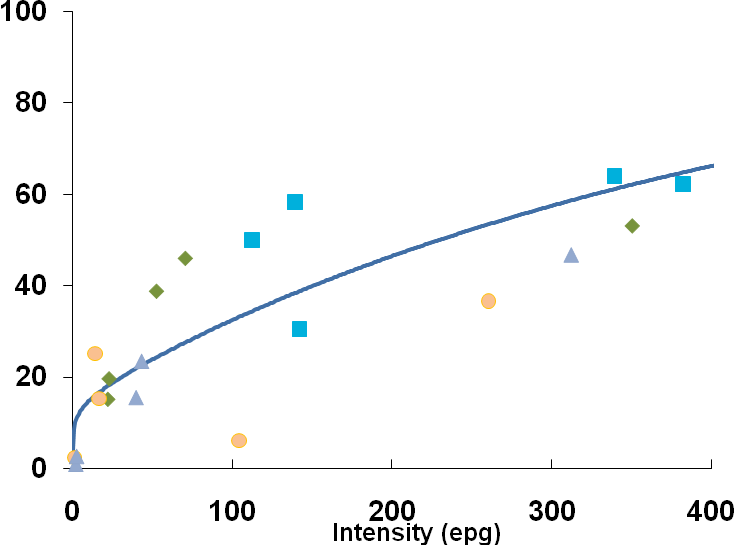

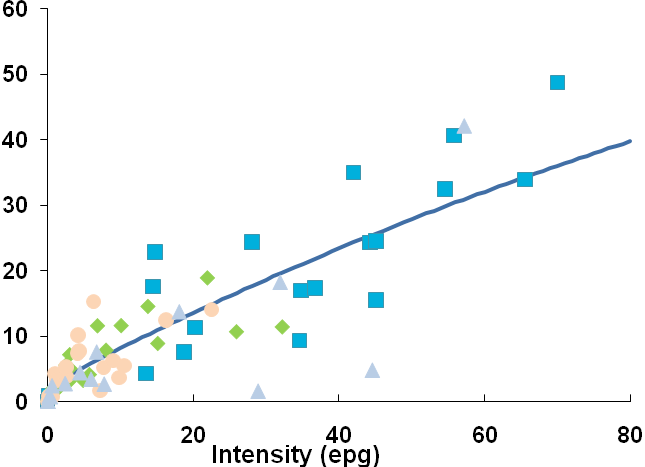


**Figure S2.** Comparison of observed population age-structure of Uganda (source: U.S. Census Bureau [3]) and the model-derived age-structure fit assuming a constant death rate.


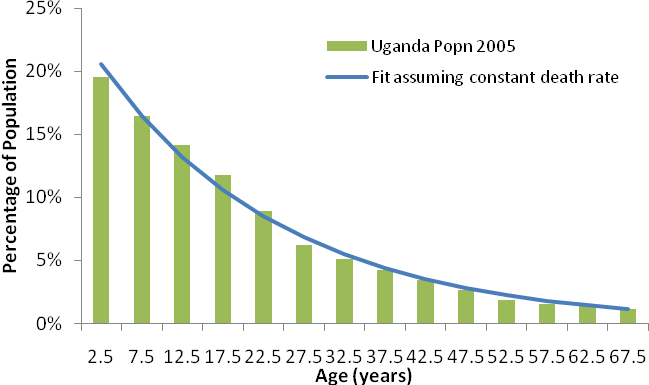


**References**

1. Chan MS, Anderson RM, Medley GF, Bundy DAP (1996) Dynamic aspects of morbidity and acquired immunity in schistosomiasis control. Acta Trop 62: 105-117.

2. WHO (2002) Prevention and control of schistosomiasis and soil-transmitted helminthiasis. Geneva: World Health Organization. i-v1 p.

3. United States Census Bureau (2005). U.S. Census Bureau International Data Base. http://www.census.gov/ipc/www/idb/country.php

4. Anderson RM, May RM (1991) Infectious Diseases of Humans: Dynamics and Control. Oxford: Oxford University Press.

5. Kumar V, Gryseels B (1994) Use of praziquantel against schistosomiasis: a review of current status. Int J Antimicrob Agents 4: 313-320.
