# Supplementary material for: Observed Reductions in Schistosoma mansoni Transmission from Large-Scale Administration of Praziquantel in Uganda: A Mathematical Modelling Study
Source: PLoS Negl Trop Dis. 2010 Nov 23;4(11):e897. doi: 10.1371/journal.pntd.0000897 (PMC2990705; doi:10.1371/journal.pntd.0000897)
Supplement: Table S1 — Parameter definitions and values used in the model. The table differentiates between parameters that were fixed throughout and those that were fitted using baseline cross-sectional and longitudinal cohort data. H = Areas of high average intensity at baseline (≥400epg), M = Areas of medium average intensity at baseline (100–399 epg), L = Areas of low average intensity at baseline (1–99epg). (0.07 MB DOC) [file pntd.0000897.s006.doc]

| **Parameter** | **Symbol** | **Units** | **Value [sensitivity analysis] or (95% CI)** | **Fixed or fitted parameter** | **Source** |
| --- | --- | --- | --- | --- | --- |
| Overdispersion parameter as a function of mean intensity | $k_{0}$ | Dimensionless | H: 0.091; M: 0.048; L: 0.001 | Fitted, Figure S1 | Protocol S1 |
| $k(M)=k_{0}+k_{1}M^{k_{2}}$ | $k_{1}$ | Dimensionless | H: 0.0019; M: 0.0032; L: 0.0192 | Fitted, Figure S1 | Protocol S1 |
|  | $k_{2}$ | Dimensionless | H: 1.000; M: 1.000; L: 0.667 | Fitted, Figure S1 | Protocol S1 |
| Human mortality rate | $\mu_{H}$ | Year^–1^ | 0.0441 | Fitted, Figure S2 | [3] |
| Worm lifespan | $1/\mu_{M}$ | Years | 4 [2, 10] | Fixed | [4] |
| Drug efficacy | $\varepsilon$ | Dimensionless | 0.95 [0.90, 0.99] | Fixed | [5] |
| Baseline *FOI* | $\lambda_{B}$ | Year^–1^ |  | Fitted | Estimated in this paper |
| Ratio of *FOI* following PZQ treatment round *P* relative to baseline (*P* = 1, 2, 3) | $\zeta_{P}$ | Dimensionless |  | Fitted | Estimated in this paper |
| Shape parameter of the age exposure profile (contact function, equation [S1]) | $\beta$ | Year^–1^ | H: 0.052 (0.041, 0.113) M: 0.056 (0.039, 0.097) L: 0.040 (0.011, 0.065) | Fitted | Estimated in this paper |
| Shape parameter of the age exposure profile (contact function, equation [S1]) | $c$ | Years | H: 1.95 (0.37, 6.54) M: 4.30 (0.73, 6.93) L: 0.164 (0, 5.93) | Fitted | Estimated in this paper |
